# Formulation of Modified-Release Bilayer Tablets of Atorvastatin and Ezetimibe: An *In-Vitro* and *In-Vivo* Analysis

**DOI:** 10.3390/polym14183770

**Published:** 2022-09-09

**Authors:** Iqra Mubeen, Muhammad Zaman, Muhammad Farooq, Asim Mehmood, Fahad Khan Azeez, Wajiha Rehman, Sohail Akhtar, Mueen Ahmad Chaudhry, Muhammad Hammad Butt, Qurat-ul-ain Shamim, Sherjeel Adnan, Muhammad Rizwan Khan, Atta-ur-Rehman

**Affiliations:** 1 Department of Pharmaceutics, Faculty of Pharmacy, The University of Lahore, Lahore 54000, Pakistan; 2 Faculty of Pharmacy, University of Central Punjab, Lahore 54000, Pakistan; 3 Department of Health Informatics, Faculty of Public Health and Tropical Medicine, Jazan University, Jazan 45142, Saudi Arabia; 4 Health Informatics Department, College of Public Health and Health Informatics, Qassim University, Buraydah 52385, Saudi Arabia; 5 Allied College of Health Sciences, Multan 59300, Pakistan; 6 Department of Medicinal Chemistry, Faculty of Pharmacy, Uppsala University, 75123 Uppsala, Sweden; 7 Department of Pharmacy, Imran Idrees College of Pharmacy, Sialkot 51040, Pakistan; 8 Department of Pharmacy, Forman Christian College, Lahore 54000, Pakistan

**Keywords:** bilayer tablets, ezetimibe, atorvastatin, HPMC K-100

## Abstract

The objective of this work was to formulate co-loaded bilayer tablets containing ezetimibe (EZB) and atorvastatin (ATC). ATC loaded in the immediate-release (IR) layer is an HMG CoA reductase inhibitor, while EZB, added in the sustained-release (SR) layer, is a lipid-lowering agent. This study was conducted to evaluate the effects of polymer on the formulation and characterization of bilayer tablets, as well as the therapeutic impact of the concurrent use of both drugs having a sequential release pattern. To obtain the optimized results, four different formulations with variable compositions were developed and evaluated for different parameters. The drug release studies were carried out using a type II dissolution apparatus, using phosphate buffer solution (PBS) of 1.2 pH for IR of EZB for an initial 2 h, followed by 24 h studies for ATC in PBS 6.8 pH. The IR layer showed rapid drug release (96%) in 2 h, while 80% of the ATC was released in 24 h from the SR layer. Locally obtained, 6-week-old female albino rats were selected for in vivo studies. Both preventive and curative models were applied to check the effects of the drug combination on the lipid profile, atherosclerosis and physiology of different organs. Studies have shown that the administration of both drugs with different release patterns has a better therapeutic effect (*p* < 0.05), both in preventing and in curing hyperlipidemia. Conclusively, through the sequential release of ATC and EZB, a better therapeutic response could be obtained.

## 1. Introduction

Patients who suffer from atherosclerotic cardiovascular disease (ASCVD), especially those with acute coronary syndrome (ACS), are at very high risk of cardiovascular disease. The risk of ASCVD can be minimized with the use of statins by reducing the low-density lipoprotein cholesterol level (LDL-C), but with the sole use of statins, some patients need additional lipid-lowering therapy to achieve the optimal level of LDL-C (<70 mg/dL). Thus, for these patients, a combination therapy should be used to achieve the desired results. When ezetimibe is combined with statins, it provides a further 17–23% reduction in LDL-C levels because it is a cholesterol absorption inhibitor [1].

Ezetimibe and atorvastatin calcium are antihyperlipidemic agents that have been developed in combination and administered for the treatment of hyperlipidemia. EZB exerts its function through the inhibition of the intestinal absorption of biliary and dietary cholesterol [2], while atorvastatin calcium acts as an HMG-CoA reductase inhibitor [3]. Their combination permits the dual inhibition of cholesterol production and absorption.

The main purpose of these current modifications in new drug delivery systems is to improve the safety and efficacy of drugs by fabricating/formulating a dose form that is easy to administer and has greater patient compatibility [4]. Ezetimibe is a lipid-lowering drug that belongs to BCS class II. It is a white powder and crystalline in nature and is freely soluble in methanol, ethanol and acetone, but is not soluble in distilled water [5]. The melting point is 163 °C with stable ambient heat. The biological half-life of ezetimibe is approximately 22 h. Its bioavailability is not affected by the concomitant administration of food, statins (HMG-CoA reductase inhibitors) and antacids [6]. Ezetimibe is a lipid-lowering agent that inhibits almost 54% of cholesterol absorption, which contributes to reducing LDL-C by almost 18–20%, but it does not cause any increase in triglyceride concentration; it also reduces the risk of cardiovascular diseases and stroke. The normal maintenance dose of ezetimibe is 10 mg/day, but in the case of patients with patients with renal impairment, the dose can be increased to 20 mg/day, under close monitoring. Atorvastatin is an HMG CoA reductase inhibitor or “statin”. It reduces the levels of low-density lipoproteins (LDL) and triglycerides in the blood, while increasing levels of high-density lipoproteins (HDL). The half-life of atorvastatin is approximately 7 h; its bioavailability is 12% after oral administration and plasma protein binding is approximately >98%. Statins are used for the treatment of hypercholesterolemia; the maintenance dose of atorvastatin is 10 mg/day. After considering the above factors of ezetimibe and atorvastatin, an attempt was made to formulate a bilayer tablet to provide an immediate-release effect and sustained-release effect, reduce the dose frequency and avoid unpredictable results of drug release [6]. The atorvastatin drug was used as an immediate-release layer and ezetimibe was used as a sustained-release layer. HPMC K-100 and Eudragit L-100 were used for the sustained-release effect—Eudragit L-100 was used because of its inertness and non-toxic behavior. The wet granulation method was used to formulate the bilayer tablets [7].

## 2. Materials and Methods

### 2.1. Materials

Ezetimibe and atorvastatin were used as APIs; HPMC K-100, PVP-30, Avicel PH 102, citric acid, sodium bicarbonate and cross povidone were gifted by CCL Pharmaceuticals, Lahore, Pakistan. Ethanol, methanol and distilled water were taken from the research laboratories of the University of Lahore, Pakistan.

#### Experimental Animals

Locally bred 6-week-old female albino rats having a weight range of 150–200 g was purchased from the Animal House Faculty of Pharmacy, the University of Lahore. All the animals were divided into their respective groups and kept at room temperature (25 ± 1 °C), with relative humidity of 45–50% and a 12 h dark/light alternate cycle. The animals had free access to clean water and standard diet *ad libitum*. Experimental animals were kept under fasting conditions before the start of the experiment for approximately 12–18 h. All experiments were conducted according to the guidelines published by the Ethics Committee of the University of Lahore, Lahore, Pakistan. 

### 2.2. Methods

#### Preparation of Bilayer Tablets

The formulations consisting of ATC as the IR layer were created with different ingredients, including citric acid, cross povidone and sodium bicarbonate (Table 1), and those with EZB as the SR layer were composed of HPMC K-100, Eudragit L-100, PVP k-100, Mg-stearate and Avicel pH 102 (Table 2) [8]. Initially, all the ingredients for the SR layer were weighed accurately and placed in a pestle and mortar and mixed for 20 min to achieve uniform mixing. During continuous mixing, a few drops of ethanol were added to produce a thick paste, followed by the formation of granules by passing it through a #30 sieve. The prepared granules were poured into a petri dish and placed in a pre-heated oven to dry. The dried granules were coated with Eudragit L-100 in a coating pan and dried again. Finally, the granules were punched using a single punch machine to produce a tablet. The compressed tablets were than coated with the IR layer having a 10 mg dose of ATC and left to dry [9]. 

### 2.3. Evaluation of ATC/EZB Bilayer Tablets

The tablets prepared with variable concentrations of excipients were evaluated in terms of different parameters to obtain a suitable formulation having the desired outcomes.

#### 2.3.1. Thickness

Uniformity in thickness and diameter is a predictive parameter of weight uniformity, which ultimately leads to uniformity of the content. Tests were performed on 10 bilayer tablets from each formulation by using a digital Vernier caliper, and mean ± standard deviation values were noted. 

#### 2.3.2. Hardness 

The force that is required to crush the tablet is considered the hardness of the tablet. Ten tablets were selected from each formulation and hardness was measured using a Monsanto hardness tester. The outcomes were recorded as mean ± standard deviation.

#### 2.3.3. Weight Variation Test 

Twenty tablets were taken from each formulation and their average weight was recorded for the purpose of identifying weight variation among formulations, and values were compared to the standard range that is mentioned in the USP Pharmacopoeia. The weight variation was calculated as a percentage. 

#### 2.3.4. Friability 

Twenty randomly pre-weighed tablets were selected for use in the friabilitor. The friabilitor was operated for 4 min at 25 rpm. After 100 revolutions, tablets were again de-dusted and weighed. The percentage of weight loss was calculated and percentage friability was recorded. 

#### 2.3.5. In Vitro Drug Release 

The dissolution studies were performed using a USP type II dissolution apparatus, firstly for 2 h in acidic media at 1.2 pH, followed by 24 h in basic media at 6.8 pH, to determine the release pattern of tablets in both media for IR and SR layers of tablets [10].

#### 2.3.6. Drug–Excipient Compatibility 

FTIR studies were conducted to determine any type of drug–excipient interaction or chemical incompatibility. FTIR spectra of pure drug polymer and prepared formulations were recorded in the range of 500 to 4000 cm^−1^ [11].

#### 2.3.7. X-ray Diffractometry 

XRD was used to identify any changes in the structure of drugs from crystalline to amorphous; samples of drugs were analyzed by a powder X-ray diffractometer. The experimental terms and conditions were as follows: 45 kV tube voltage, 40 mA tube current and scanning angle 2θ was 5–50° [12]. 

#### 2.3.8. Thermal Gravimetric Analysis (TGA) of Bilayer Tablets 

A TGA instrument (Pyris diamond series TGA/DTA, Shelton, Connecticut, United States) was used to obtain TGA curves of API, NPI and formulated bilayer tablets by placing almost 6–7 mg of sample on an alumina crucible. The heating rate was 10 °C/min with the range of 40–1000 °C, under an atmosphere of nitrogen with a 120 mL/min flow rate. The TGA cell was calibrated with indium (156 °C) and tin (232 °C). Thermogravimetric analysis of the pure drugs, polymer and optimized formulation of bilayer tablets was performed.

#### 2.3.9. Differential Scanning Calorimetry 

A DSC test was used to measure the energy required to keep the sample and reference both at the same temperature. Thermograms of both drugs, (ezetimibe and atorvastatin) and bilayer tablets were acquired by using SDT (Q-600, thermal analyzer, Shimadzu, Kyoto, Japan). The DSC test was applied to identify any chemical interactions between ezetimibe, atorvastatin and HPMC K-100. In this method, a drug and polymer mixture are heated in an aluminum pan, with a flow rate of 10 °C in the range of 0 to 300 °C. Nitrogen flow was kept at 40 mL/min, and 4–8 mg of the sample was placed in the aluminum pan. Samples were assessed three times to check the reliability of results. 

### 2.4. Animal Selection 

Six-week-old female rats were assigned to 1 of 7 groups, each consisting of 3 rats. All rats were fed with a general diet and HC (2% cholesterol in coconut oil) for 4 weeks, except group 1. Group 1, the control group, was fed only a standard diet and had free access to drinking water *ad libitum*. Group 2 was the negative control group [13]. The test groups, Groups 3 and 4, were treated concomitantly and administered EZB and ATC tablets and the standard marketed drug through oral gavage. Treatment among test groups was given after 2 h of HC diet. The solvent (D.W) was given to Groups 1 and 2, through oral gavage, for the same experimental period. This preventive study was completed after one month. In the curative model, Group 5, the negative control, was fed only the standard diet and drinking water. Groups 6 and 7 were treated similarly to Groups 3 and 4. The same dose was given for one month and body weight was measured weekly [14]. 

#### 2.4.1. Dose and Dose Frequency

All the treated animals received a single dose of EZB and ATC at a dose of 3 mg/kg/day in the form of suspension and marketed drug in oral solution form.

#### 2.4.2. Collection of Blood Samples

At the end of the experimental period, the animals were anesthetized, and blood was drawn from the animals after cardiac puncture for the evaluation of the lipid profile (HDL, VLDL, LDL, CH, TG).

#### 2.4.3. Determination of Plasma Lipids

Plasma cholesterol levels were determined by using a commercial kit, ab65390. High-density lipoprotein (HDL), total cholesterol, cholesterol and triglyceride (TG) levels were measured. The LDL cholesterol level was calculated by using the Friedewald formula and very low-density lipoprotein (V-LDL) was calculated by using the ratio of TG/5 [15].

#### 2.4.4. Histopathological Analysis

Aorta was removed and selected for histopathological study. Sections were stained with hematoxylin and eosin (H&E) and examined under a light microscope [16].

## 3. Results and Discussion

### 3.1. Evaluation of ATC/EZB Bilayer Tablets

The prepared tablets were evaluated for post-compression studies (Table 3) and the diameters of all tablets prepared from four formulations ranged from 7.01 ± 0.052 to 7.02 ± 0.104, while the thickness (7.01 ± 0.076 to 7.01 ± 0.09), hardness (9.8–10.1 ± 0.10 to 10.9–11.2 ± 0.012) and friability (0.21 ± 0.005 to 0.37 ± 0.12) were also measured. All tablet formulations fell within the official limit of the British Pharmacopeia specified for weight variation. The formulation F1 showed the maximum drug content (104.15%), while F3 showed the least (99.8%). 

### 3.2. In Vitro Drug Release Studies

**Figure 1:**
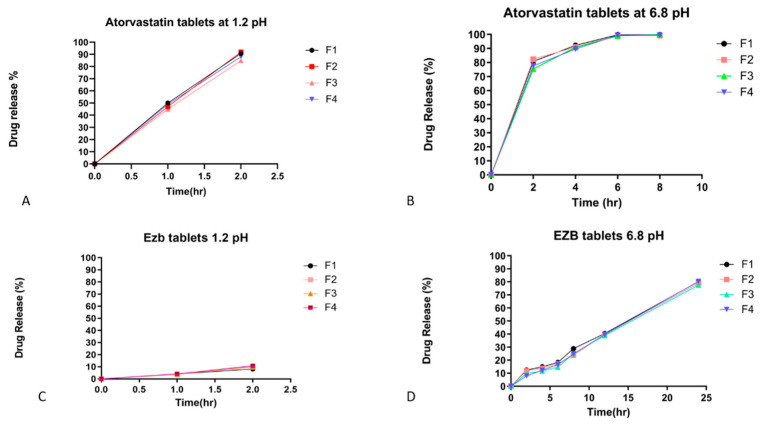
Drug release of bilayer coated (Eudragit L-100) tablets of ezetimibe and atorvastatin at pH 1.2 and pH 6.8. Atorvastatin release in stomach at pH 1.2 and at intestinal pH 6.8 (**A**,**B**), with slow release after 2 h stay. EZB release and stay in stomach at pH 1.2 is minimum and intestinal pH 6.8 showed rapid release and stay for 24 h (**C**,**D**).

The dissolution was carried out by placing a tablet in the dissolution vessel containing 900 mL of dissolution medium (buffer 1.2 pH). After adjusting the temperature to 37 °C, tablets were transferred and continuously stirred at 50 rpm. Then, 5 mL of sample was withdrawn after a specified time interval at 1 h and 2 h, and then transferred to the pH 6.8 phosphate buffer solution and subjected to the same procedure for 4 h, 6 h, 8 h and 24 h. This procedure was used for the determination and evaluation of atorvastatin calcium in acidic media for the immediate-release layer, and the next phase for the sustained-release layer of ezetimibe. The amount of drug released was determined and analyzed by adjusting the wavelengths of the drugs and calculating their absorbance. Results showed that the drug release of the atorvastatin calcium layer showed 51.93% release within one hour and 96.29% in the second hour (Figure 1). The SR layer showed only 8.9% release of the drug in acidic media in two hours. In basic media, drug release was 80% until the 24th hour. The results demonstrated the SR effect of EZB [17]. Bilayer tablets providing the immediate release and sustained release of rosuvastatin and atenolol were prepared by M. A. Elsayed. Immediate-release bilayer tablets (92.34 ± 2.27) acted within 60 min at a low pH of 1.2, under the same conditions as for the sustained-release tablets (96.65 ± 3.36% within 12 h) [18]. The EZB and ATC bilayer tablets formulated here exhibited comparable efficacy with previously formulated bilayer tablets. 

### 3.3. Chemical Compatibilities 

**Figure 2:**
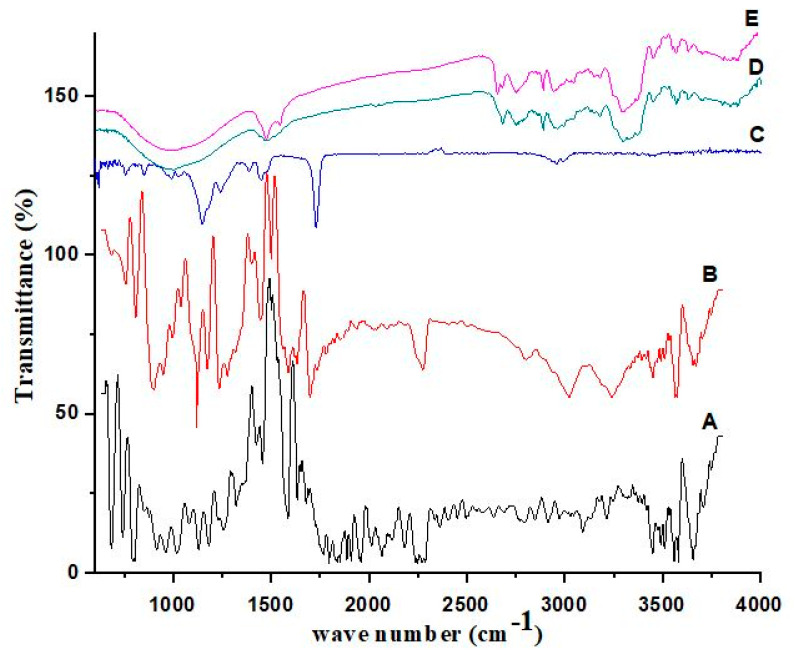
FTIR spectra of atorvastatin (A), ezetimibe (B), HPMC K-100 (C), formulation F1 (D) and F2 (E).

The FTIR spectrum (Figure 2) of atorvastatin calcium (A) showed a peak at 1750 cm^−1^ indicating the presence of a C=O stretching peak of carboxylic acid. The peak present at 1120 cm^−1^ indicated C–F stretching and that at 3270 cm^−1^ indicated O–H stretching, while the peak at 3350 cm^−1^ confirmed the presence of stretching due to N–H, the aliphatic primary amine group. The FTIR spectrum of ezetimibe (B) showed a wide band at 1160 cm^−1^ confirming the presence of a fluorine group via the stretching of C–F. The peak present at 1750 cm^−1^ reflected C=O stretching, indicating the presence of carboxylic acid; the peak at 3214 cm^−1^ indicated the stretching due to O–H, an alcoholic group. The peak at 3474 cm^−1^ showed stretching due to N–H, an amine group. In the spectrum of HPMC K-100 (C), a peak at 1262 cm^−1^ indicated the presence of the C–O stretching peak of the aromatic ester group. The peak at 1750 cm^−1^ showed the presence of C–H stretching, which indicated aromatic compounds. The FTIR spectrum of formulation F1 (D) showed a peak at 3251 cm^−1^ due to O–H stretching and a peak at 3454 cm^−1^ confirmed the stretching due to N–H, an aliphatic primary amine group. The FTIR spectrum of formulation F2 (E) showed a peak at 3260 cm^−1^ due to O–H stretching and a peak at 3504 cm^−1^ confirming the stretching due to N–H, an aliphatic primary amine group [19]. 

### 3.4. X-ray Powder Diffraction Analysis of Bilayer Tablets 

**Figure 3:**
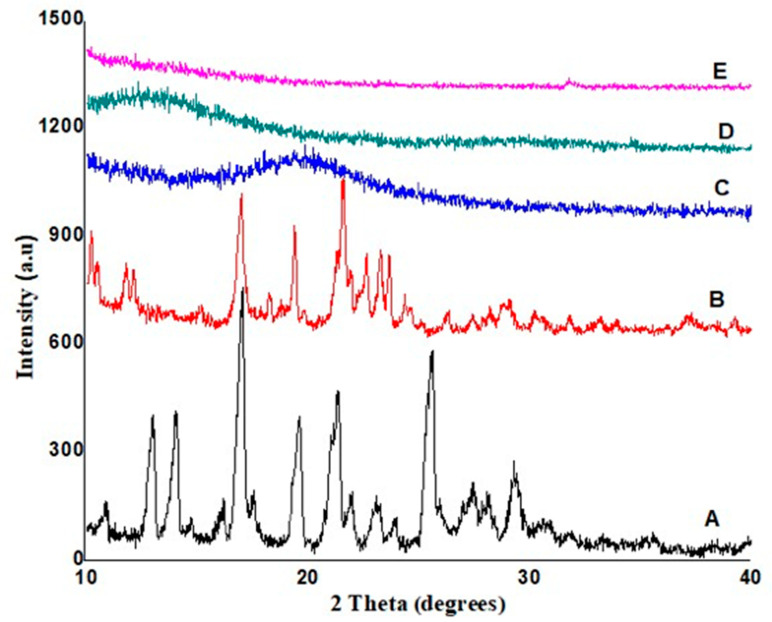
XRD of atorvastatin (A), ezetimibe (B), HPMC K-100 (C) formulation F1 (D) and F2 (E).

The XRD pattern of atorvastatin exhibited intense peaks at diffraction angles (2θ) of 13.007°, 14.04°, 17.03°, 19.59°, 21.339°, 25.6° and 29.28°, and their corresponding counts were 442, 450, 798, 438, 509, 623 and 314. The XRD pattern of ezetimibe exhibited intense peaks at diffraction angles (2θ) of 10.2°, 11.78°, 16.9°, 19.36°, 21.54°, 22.63°and 23.24°, and their corresponding counts were 323, 233, 427, 340, 471, 262 and 272. HPMC K-100 showed low-intensity peaks at diffraction angles (2θ) of 10.28°, 11.30°, 13.33°, 16.09°, 18.89°, 19.87° and 25.56°, and their corresponding counts were 231, 214, 198, 196, 221, 254 and 107. Atorvastatin ezetimibe co-loaded bilayer tablets’ diffractogram showed low-intensity peaks at diffraction angles (2θ) of 10.02°, 11.54°, 13°, 17.23°, 19.55°, 20.03° and 28.59°, and their corresponding counts were 196, 191, 196, 174, 198, 224 and 197. For another formulation, the atorvastatin ezetimibe co-loaded bilayer tablets’ diffractogram showed low-intensity peaks at diffraction angles (2θ) of 10.12°, 11.48°, 13.02°, 17.25°, 19.61°, 20.28° and 28.35°, and their corresponding counts were 187, 190, 206, 177, 167, 163 and 133; as compared to the pure drugs, these values revealed a decrease in the crystalline nature of both drugs (Figure 3). Sharp peaks for both drugs became low-intensity peaks as they were excipients and active ingredients entrapped with each other, resulting in a loss of crystallinity, and they became amorphous.

### 3.5. Thermal Gravimetrical Analysis of EZB/ATC Tablets

**Figure 4:**
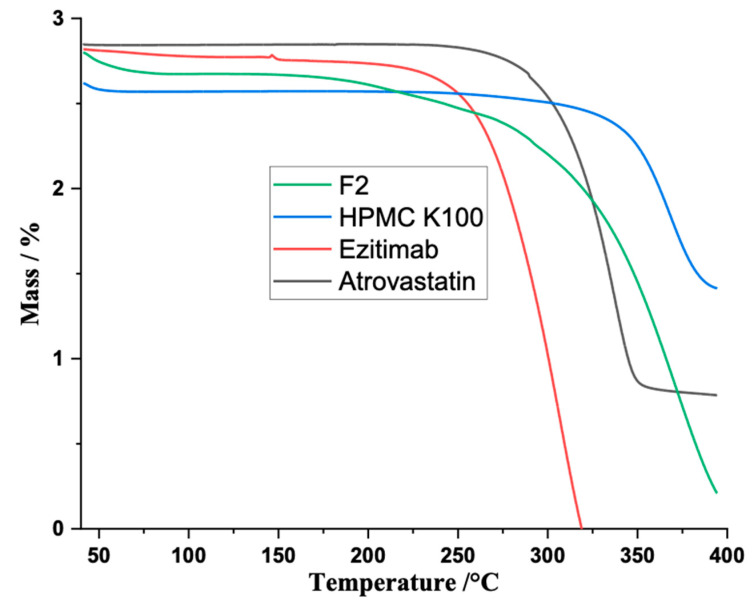
TGA of atorvastatin, ezetimibe, HPMC K-100 and formulation F2.

In TGA analysis, graph curves showed weight loss regions with the rise in temperature, as presented in Figure 4. Initially, drug loss started at 260 °C, with approximately 80% weight loss at 349 °C, while EZB showed weight loss initially at 230 °C and showed 100% weight loss at 320 °C. The polymer (HPMC K-100) showed initial weight loss at 325 °C and its weight loss reached 50% at 394 °C, while the formulation showed initial weight loss at 195 °C and approximately 89–90% weight loss at 394 °C. This indicates that the mixing of pure drugs and polymers could produce only minor changes in the melting endotherm of API and did not show an interaction or incompatibility between API and NPI [20].

### 3.6. Differential Scanning Calorimetry (DSC) of Bilayer Tablets 

**Figure 5:**
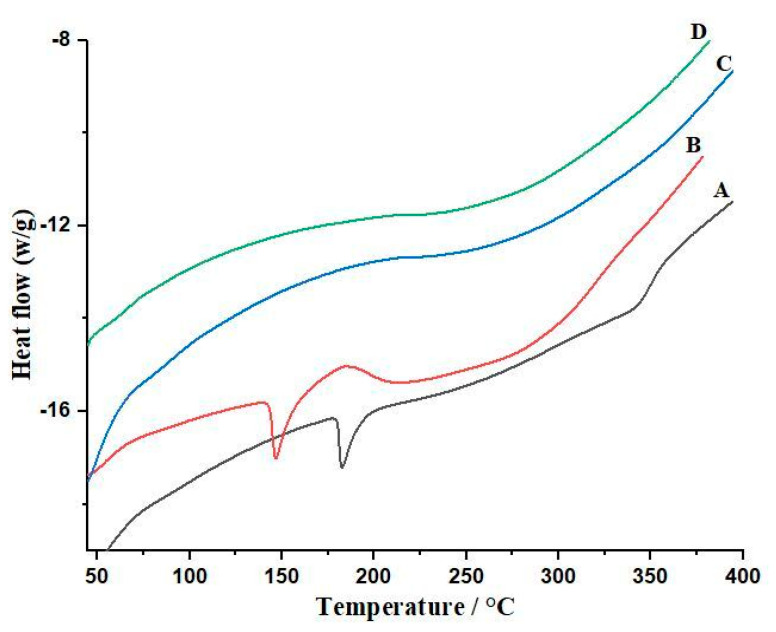
DSC of atorvastatin (A), ezetimibe (B), HPMC K-100 (C) and formulation F2 (D).

In DSC studies as presented in Figure 5, both endothermic and exothermic peaks have been observed. Atorvastatin and ezetimibe showed endothermic peaks. In our study results, the curves showed that the MP of atorvastatin was 150–200, and it was 100 to 150 for ezetimibe. Our results were similar to those of reported studies where ATC showed an endothermic peak at 182 °C [21], while EZB showed an endothermic peak at 146 °C [22]. In our study, the results were in line with the melting points of the pure drugs. The DSC curve of formulation F2 did not show any endothermic peak, indicating the polymer and drug’s compatibility. No interaction was observed in the DSC curves of both drugs and polymer, and the literature also reported the same phenomenon, where no change was observed in the formulation [23]. 

### 3.7. Pharmacodynamic Studies 

Pharmacodynamics has been carried out in two phases of the study: the first one was preventive, and the second one was a curative study. Four groups of animals were evaluated: the first one was the normal group (no disease induction), the second group was a negative control (disease induced without treatment), the third group received EZB/ATC tablets (disease induced and treated with newly formed bilayer coated tablets) and the fourth group received EZB/ATC standard drug (disease induced treated with marketed available dosage form). 

#### 3.7.1. Effectiveness of Drug Treatment in Preventive Studies 

##### Lipid Profile in Normal vs. Negative Control Group

**Figure 6:**
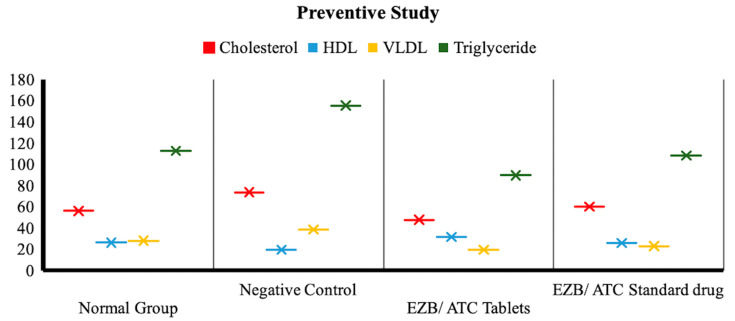
The effects of drugs on cholesterol, HDL, VLDL and triglycerides during preventive treatment in normal group, negative control, EZB/ATC tablet group and EZB/ATC standard drug group.

The cholesterol level, HDL and VLDL showed a non-significant increase when compared with the negative control group, whereas there was a highly significant (*p* < 0.001) increase in triglyceride levels (Figure 6).

##### Lipid Profile in Negative Control Group vs. Treated Groups

The cholesterol level, HDL and VLDL showed a non-significant increase when compared to the negative control group when treated with film, granules and the standard drug of atorvastatin/ezetimibe, whereas a highly significant (*p* < 0.001) increase in triglyceride levels was observed in all treated groups, as shown in Figure 6 and Table 4.

##### Effect on Rats’ Vital Organs

After the dissection of all rats in the preventive study, the different organs of animals were weighed and compared with those in the negative control group. The effect of treatment was significant on all vital organs’ weight, width, length and volume values, compared to the negative control group (Table 5).

#### 3.7.2. Drug Treatment and Its Effects in Curative Model

In the curative phase, selected rats’ cholesterol levels were already high, and treatment was given to rats for one month only. The body weight of all groups was checked on a weekly basis; after completion of the study, necropsy was performed. The values of plasma lipoprotein were recorded and histopathology of the aorta was performed.

##### Plasma Lipoprotein Analysis in Curative Phase

Rats were dissected after 8–10 h fasting. Blood samples were taken to check the total plasma cholesterol, HDL, VLDL and triglycerides by using commercial enzymatic assays. Results of different parameters in all groups are shown in Table 6. 

##### Lipid Profile in Normal vs. Negative Control Group 

The cholesterol level showed a significant effect (*p* < 0.05) when compared to the normal group and the negative control group, while triglycerides showed a highly significant (*p* < 0.001) effect. HDL and VLDL showed non-significant results. 

##### Lipid Profile in Normal vs. Negative Control Group in Curative Phase

**Figure 7:**
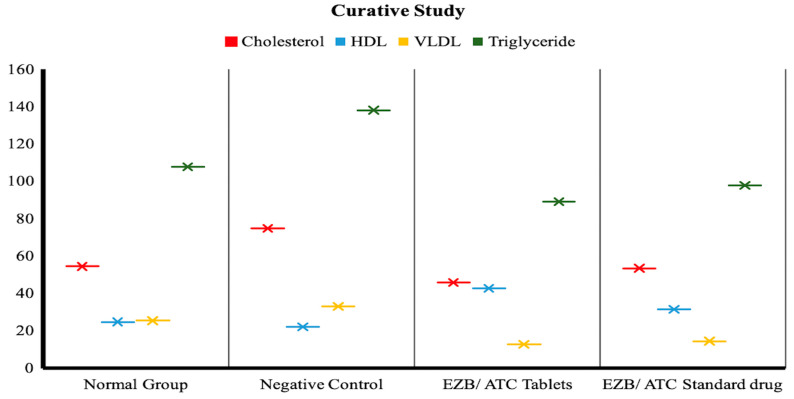
The effects of drugs on cholesterol, HDL, VLDL and triglycerides during curative treatment in normal group, negative control, EZB/ATC tablet group and EZB/ATC standard drug group.

The cholesterol level (significant *p* < 0.05) and triglycerides, when compared with the negative control group, were highly significant (*p* < 0.001) in the group receiving atorvastatin/ezetimibe tablets. Meanwhile, the standard drug group showed a significant effect on cholesterol (*p* < 0.05) and a non-significant increase in HDL. A significant (*p* < 0.05) increase was observed in VLDL for all treated groups (Figure 7).

##### Effect on Rats’ Vital Organs in Curative Phase

After the dissection of all rats in the preventive study, the different organs of animals were weighed and compared with those in the negative control group. The effect of treatment was significant on all vitals organs’ weight, width, length and volume values, compared to the negative control group. Results are shown in Table 7. 

### 3.8. Histopathological Evaluation

**Figure 8:**
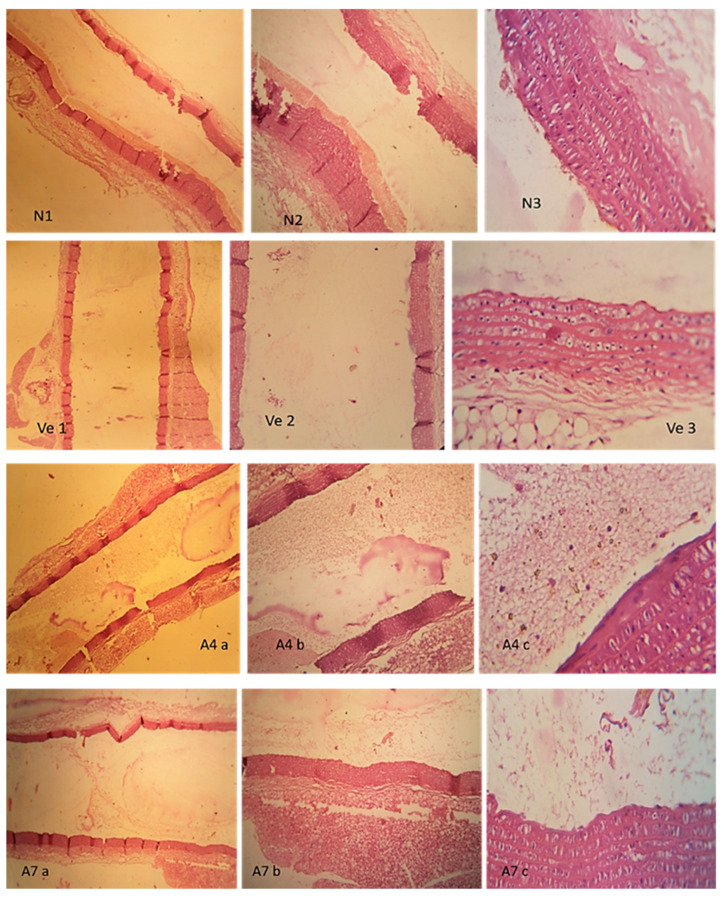
Histopathological slides of aorta obtained during comparative analysis of *control group* (**N 1–3**), where uniform intact layer of endothelial lining; *negative control group* (**Ve 1–3**) where endothelial cell layer is normal. In *tablet (**A4 a–c**) formulation group* given to animal the intact endothelial lining with uniform tunical media and adventia, and in *marketed drug (**A7 a–c**) group* intact endothelial lining with uniform tunical media and adventia. However, in all groups no sign of atherosclerosis or necrosis were seen.

The aorta was selected from all groups as presented in Figure 8. We washed it with normal saline and then preserved it in 10% formalin solution for histopathological investigation. Tissues of aorta were stained with hematoxylin and eosin for evaluation. Images of the aorta from all groups were taken at different magnification powers (40X, 100X and 400X), with the results shown in Table 8.

## 4. Conclusions

Bilayer layer tablets were prepared, including immediate- and sustained-release layers, by the wet granulation method using HPMC K-100 and two APIs (ezetimibe and atorvastatin). Compressibility and blending evaluations on the basis of optimization methods showed that the bilayer tablets met acceptance criteria in all aspects. Drug release from the sustained-release layer was 8.9% in two hours in acidic media and revealed zero-order kinetics. The drug release from the immediate-release layer amounted to 96.29% and displayed first-order kinetics. The developed formulations were applied to albino rats and compared to the drug marketed with the same ingredients, and there were no signs or symptoms of atherosclerosis. This bilayer formulation provides several advantages over the marketed drug in terms of the simple manufacturing process and cost-effectiveness.

## Figures and Tables

**Table 1:** Composition of immediate-release layer.

Citric Acid (mg)	Cross Povidone (mg)	Sodium Bicarbonate (mg)
24.50	48.65	83.50
28.50	44.65	83.50
32.50	40.65	83.50
36.50	36.65	83.50
*Constant quantity (10 mg) of EZB was used in all the formulations*		

**Table 2:** Composition of sustained-release layer.

Polymer (mg)	Binder (mg)	Glidant (mg)	Diluent (mg)
40	16	2	132
20	15	2	153
68	12.5	2	107.50
60	15	2	113
*Constant quantity (10 mg) of EZB was used in all the formulations*			

**Table 3:** Post-compression studies of bilayer tablets.

Post-Compression Studies	F1	F2	F3	F4
Diameter (mm)	7.02 ± 0.104	7.01 ± 0.109	7.01 ± 0.052	7.01 ± 0.063
Thickness (mm)	7.01 ± 0.08	7.01 ± 0.09	7.01 ± 0.076	7.01 ± 0.077
Weight Variation (%)	Within Limit (±7.5%)	Within Limit (±7.5%)	Within Limit (±7.5%)	Within Limit (±7.5%)
Hardness (N)	10.9–11.2 ± 0.012	9.8–10.1 ± 0.10	10.4–10.9 ± 0.23	10.8–11.2 ± 0.132
Friability (%)	0.26 ± 0.007	0.21 ± 0.005	0.36 ± 0.07	0.37 ± 0.12
Drug Content (%)	102.04 ± 2.06	101.09 ± 1.77	99.88 ± 0.988	104.15 ± 2.84

**Table 4:** Effect of atorvastatin and ezetimibe on lipid profile of rats in preventive study.

Parameters	Normal Group	Negative Control	EZB/ATC Tablets	EZB/ATC Standard Drug
Triglyceride	113 ± 14.00	155.66 ± 31.19	90.00 ± 25.12	108.66 ± 16.07
VLDL	28.33 ± 6.35	39.00 ± 3.606	20.00 ± 5.19	23.33 ± 9.29
HDL	26.66 ± 8.50	20.00 ± 5.508	32.00 ± 3.606	26.33 ± 6.80
Cholesterol	56.33 ± 11.59	74.00 ± 11.54	48.00 ± 2.64	60.33 ± 4.04

**Table 5:** Effect of atorvastatin and ezetimibe on vital organs of rats in preventive study.

Group	Units	Liver	Spleen	Heart	Kidneys	
					L	R
Normal Group	Weight (g)	0.760 ± 0.010	8.7466 ± 1.017	0.953 ± 0.104	1.0233 ± 0.32	1.0233 ± 0.32
	Width (cm)	3.066 ± 0.115	5.2666 ± 0.321	1.246 ± 0.12	1.233 ± 0.153	1.233 ± 0.153
	L (cm)	0.966 ± 0.058	3.800 ± 0.200	1.233 ± 0.058	1.933 ± 0.058	1.933 ± 0.058
	Vol (mL)	0.556 ± 0.075	5.720 ± 0.491	0.733 ± 0.035	0.570 ± 0.026	0.570 ± 0.026
Negative Control	Weight (g)	0.846 ± 0.025	8.8333 ± 1.041	1.200 ± 0.10	1.3666 ± 0.153	1.3666 ± 0.153
	Width (cm)	3.866 ± 0.153	5.7666 ± 0.153	1.500 ± 0.10	1.313 ± 0.032	1.313 ± 0.032
	L (cm)	1.200 ± 0.100	3.8333 ± 0.115	1.690 ± 0.101	2.200 ± 0.100	2.200 ± 0.100
	Vol (mL)	0.820 ± 0.130	6.666 ± 0.473	0.956 ± 0.055	0.840 ± 0.036	0.840 ± 0.036
EZB/ATC Tablets	Weight (g)	0.343 ± 0.049	4.6633 ± 0.386	0.530 ± 0.030	0.420 ± 0.020	0.420 ± 0.020
	Width (cm)	2.333 ± 0.153	4.200 ± 0.265	0.483 ± 0.029	0.906 ± 0.012	0.906 ± 0.012
	L (cm)	0.653 ± 0.047	2.9333 ± 0.058	0.656 ± 0.051	1.366 ± 0.058	1.366 ± 0.058
	Vol (mL)	0.290 ± 0.010	3.4533 ± 0.423	0.376 ± 0.040	0.386 ± 0.045	0.386 ± 0.045
EZB/ATC Standard Drug	Weight (g)	0.386 ± 0.023	4.4533 ± 0.528	0.536 ± 0.057	0.430 ± 0.020	0.430 ± 0.020
	Width (cm)	2.366 ± 0.252	4.100 ± 0.173	0.486 ± 0.081	0.866 ± 0.058	0.866 ± 0.058
	L (cm)	0.766 ± 0.058	2.9333 ± 0.058	0.780 ± 0.020	1.400 ± 0.100	1.400 ± 0.100
	Vol (mL)	0.296 ± 0.015	3.3966 ± 0.100	0.363 ± 0.055	0.450 ± 0.030	0.450 ± 0.030

**Table 6:** Effect of atorvastatin and ezetimibe on lipid profile of rats in curative study.

Parameters	Normal Group	Negative Control	EZB/ATC Tablets	EZB/ATC Standard Drug
Triglyceride	107.66 ± 5.77	138 ± 30.199	89.00 ± 1.00	97.66 ± 6.55
VLDL	25.333 ± 1.155	33.00 ± 6.028	12.633 ± 0.577	14.33 ± 1.00
HDL	24.66 ± 8.083	22.00 ± 3.215	42.667 ± 3.055	31.333 ± 3.60
Cholesterol	54.33 ± 8.66	74.667 ± 5.77	45.66 ± 3.214	53.333 ± 4.61

**Table 7:** Effect of atorvastatin and ezetimibe on vital organs of rats in curative study.

Group	Units	Liver	Spleen	Heart	Kidneys	
					L	R
Normal Group	Weight (g)	8.7466 ± 1.017	0.673 ± 0.150	0.953 ± 0.104	1.140 ± 0.095	1.140 ± 0.095
	Width (cm)	5.2666 ± 0.321	3.566 ± 0.115	1.300 ± 0.100	1.233 ± 0.153	1.233 ± 0.153
	L (cm)	3.800 ± 0.200	0.983 ± 0.029	1.300 ± 0.173	1.933 ± 0.058	1.933 ± 0.058
	Vol (mL)	7.000 ± 0.529	0.670 ± 0.040	0.733 ± 0.035	0.570 ± 0.026	0.570 ± 0.026
Negative Control	Weight (g)	7.266 ± 0.462	0.503 ± 0.023	0.800 ± 0.087	1.027 ± 0.025	1.027 ± 0.025
	Width (cm)	3.667 ± 0.289	2.533 ± 0.025	1.033 ± 0.058	1.167 ± 0.153	1.167 ± 0.153
	L (cm)	3.200 ± 0.100	0.900 ± 0.100	1.033 ± 0.101	1.050 ± 0.050	1.050 ± 0.050
	Vol (mL)	6.390 ± 0.248	0.513 ± 0.130	0.746 ± 0.05	0.613 ± 0.050	0.613 ± 0.050
EZB/ATC Tablets	Weight (g)	4.103 ± 0.18	0.296 ± 0.068	0.440 ± 0.01	0.423 ± 0.025	0.423 ± 0.025
	Width (cm)	2.400 ± 0.100	1.840 ± 0.053	0.463 ± 0.029	0.600 ± 0.100	0.600 ± 0.100
	L (cm)	1.873 ± 0.110	0.516 ± 0.067	0.286 ± 0.051	0.740 ± 0.036	0.740 ± 0.036
	Vol (mL)	2.700 ± 0.100	0.290 ± 0.010	0.370 ± 0.030	0.390 ± 0.010	0.390 ± 0.010
EZB/ATC Standard Drug	Weight (g)	4.786 ± 0.280	0.433 ± 0.023	0.393 ± 0.006	0.650 ± 0.050	0.650 ± 0.050
	Width (cm)	2.783 ± 0.104	1.950 ± 0.050	0.553 ± 0.057	0.900 ± 0.050	0.900 ± 0.050
	L (cm)	2.166 ± 0.05	0.653 ± 0.129	0.396 ± 0.020	0.923 ± 0.025	0.923 ± 0.025
	Vol (mL)	3.696 ± 0.270	0.316 ± 0.015	0.400 ± 0.017	0.573 ± 0.032	0.573 ± 0.032

**Table 8:** Interpretation of aorta slides.

Slide No.	Photo Name	Remarks
N	N 40X N 100X N 400X	Uniform intact layer of endothelial lining, uniform thickness of tunica media and adventia. No sign of necrosis or atherosclerosis.
-Ve	-Ve 40X -Ve 100X -Ve 400X	Endothelial cell layer is normal. Uniform thickness of tunica media and tunca adventia. Localized disarrangement of the elastic tissue is observed. Overall, no sign of necrosis or atherosclerosis. The endothelial cells have basophilic pink cytoplasm.
Ve2	Ve2 40X Ve2 100X Ve2 400X	Intact endothelial lining with uniform tunical media and adventia. Occasionally, endothelial cells contained brownish pigment.
A4	A4 40X A4 100X A4 400X	Intact endothelial lining with uniform tunical media and adventia. Occasionally, endothelial cells contained brownish pigment. Brown prigmented crystals were seen in the clotted blood smear in the aotic lumen. However, no sign of atherosclerosis.
A7	A7 40X A7 100X A7 400X	Intact endothelial lining with uniform tunical media and adventia. No sign of etherosclerosis.

## Data Availability

Not applicable.
